# Familial Risk of Chronic Musculoskeletal Pain and the Importance of Physical Activity and Body Mass Index: Prospective Data from the HUNT Study, Norway

**DOI:** 10.1371/journal.pone.0153828

**Published:** 2016-04-15

**Authors:** Ragnhild Lier, Paul Jarle Mork, Andreas Holtermann, Tom Ivar Lund Nilsen

**Affiliations:** 1 Department of Public Health and General Practice, Norwegian University of Science and Technology, Trondheim, Norway; 2 Liaison Committee between the Central Norway Regional Health Authority (RHA), Stjørdal, Norway, and the Norwegian University of Science and Technology (NTNU), Trondheim, Norway; 3 National Research Centre for the Working Environment, Copenhagen, Denmark; Leibniz Institute for Prevention Research and Epidemiology (BIPS), GERMANY

## Abstract

The main objectives of the current study was i) to prospectively examine if chronic musculoskeletal pain in parents is associated with risk of chronic musculoskeletal pain in their adult offspring, and ii) to assess if these parent-offspring associations are modified by offspring body mass index and leisure time physical activity. We used data on 4,742 adult offspring linked with their parents who participated in the population-based HUNT Study in Norway in 1995–97 and in 2006–08. Family relations were established through the national Family Registry. A Poisson regression model was used to estimate relative risk (RR) with 95% confidence interval (CI). In total, 1,674 offspring (35.3%) developed chronic musculoskeletal pain during the follow-up period of approximately 11 years. Both maternal (RR: 1.26, 95% CI: 1.03, 1.55) and paternal chronic musculoskeletal pain (RR: 1.29, 95% CI: 1.06, 1.57) was associated with increased risk of offspring chronic musculoskeletal pain. Compared to offspring of parents without chronic musculoskeletal pain, the adverse effect of parental pain was somewhat stronger among offspring who reported a low (RR: 1.82, 95% CI: 1.32, 2.52) versus high (RR: 1.32, 95% CI: 0.95, 1.84) level of leisure time physical activity. Offspring of parents with chronic musculoskeletal pain and who were classified as obese had more than twofold increased risk (RR: 2.33, 95% CI: 1.68, 3.24) of chronic musculoskeletal pain compared to normal weight offspring of parents without pain. In conclusion, parental chronic musculoskeletal pain is positively associated with risk of chronic musculoskeletal pain in their adult offspring. Maintenance of normal body weight may reduce the risk of chronic musculoskeletal pain in offspring of pain-afflicted parents.

## Introduction

Recent family linkage studies have shown that parental pain is strongly associated with the prevalence of chronic musculoskeletal pain (CMP) in offspring, both during adolescence [1] and in later adulthood [2]. Although the importance of family history of CMP has been recognized for decades [3], no previous study has prospectively examined offspring risk of CMP in relation to parental pain reporting.

Inter-generational transfer of chronic musculoskeletal pain could be explained by both genetic heritability [4, 5] and shared environment [3, 6–8]. However, it has been suggested that the genetic influence is larger in more severe pain conditions, such as chronic widespread pain and pain conditions that interfere with daily activities [4, 5]. It may be speculated that offspring who carry an inherited susceptibility to develop CMP are more vulnerable to other risk factors for CMP, such as physical inactivity [9–12] and obesity [13, 14]. Prospective studies have shown that regular physical exercise and a normal body weight is associated with a reduced risk of pain in neck/shoulders [15–17], low back [18, 19], and upper limbs [20]. It may therefore be hypothesized that the adverse effect of parental CMP on risk of CMP in offspring is amplified by physical inactivity and obesity in the offspring.

In a family linkage study of parents and their adult offspring we prospectively examined the risk of offspring CMP in relation to parental pain reporting, and if these parent-offspring associations are modified by offspring body mass index (BMI) and leisure time physical activity.

## Materials and Methods

### Study population

The HUNT Study is a large population based longitudinal health study conducted within the county of Nord-Trøndelag, Norway. The study has been carried out in three consecutive surveys, first in 1984–1986 (HUNT1), then in 1995–1997 (HUNT2), and last in 2006–2008 (HUNT3). In all three surveys, all residents 20 years of age and older were invited to participate, and information on lifestyle and health related factors were collected by questionnaires and a clinical examination. Since no information on musculoskeletal pain was obtained at HUNT1, those who were eligible for inclusion in the current study had participated at either HUNT2 or HUNT3. At HUNT2, 93,898 persons were invited to participate, and 65,237 (70%) attended the study, whereas at HUNT3 93,860 were invited and 50,807 (54%) chose to participate [21, 22]. A total of 36,415 participated at both HUNT2 and HUNT3. More detailed information about the HUNT study can be retrieved from http://www.hunt.ntnu.no/edu/.

Each participant signed a written consent, and the study was approved by the Regional Committee for Medical and Health Research Ethics Central Norway (project no. 2011/1455/REC Central).

### Record linkage

**Fig 1:**
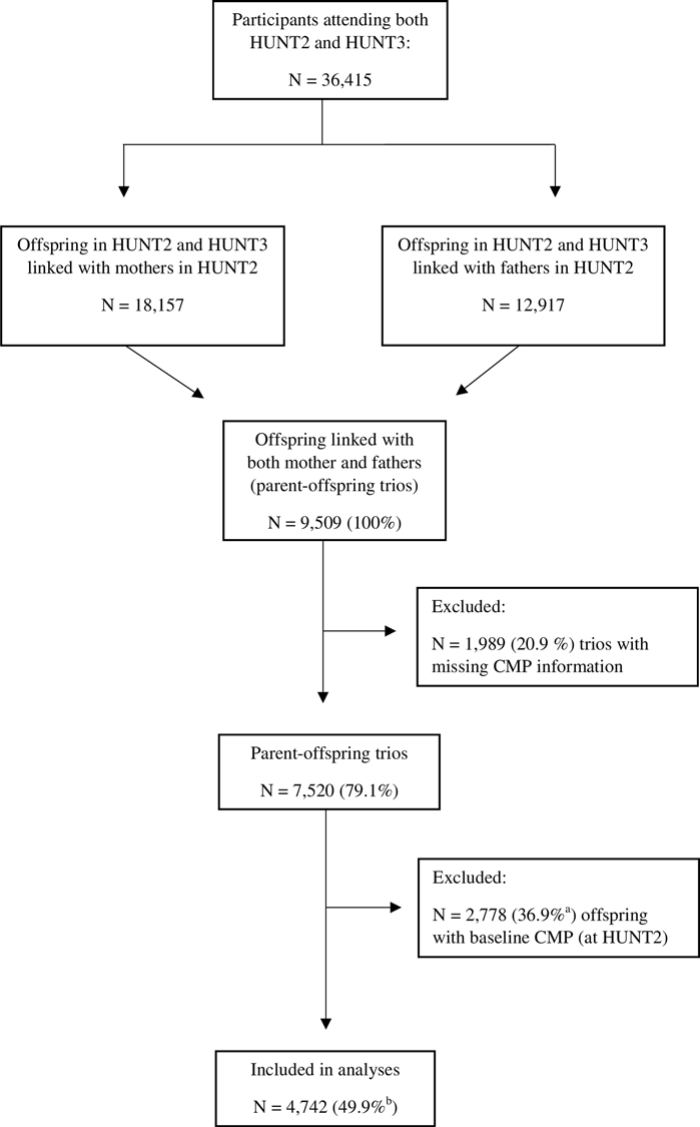
Flow chart showing selection procedures into the study. CMP: chronic musculoskeletal pain. ^a^Percentage of N = 7,520 trios. ^b^Percentage of 9,509 trios.

The unique personal identification number held by all Norwegian citizens was used to link each participant's record to information from the Family Registry at Statistics Norway, and thus establish family data of biological parents and their offspring in the HUNT Study. For the purpose of the present study we identified 9,509 parent-offspring trios where the offspring had family linkage to both their mother and father. 1,989 (20.9%) of these trios were excluded due to incomplete information about CMP. Parental information about CMP was retrieved from HUNT2.Due to the prospective nature of the data, we excluded 2,778 (36.9%) trios where offspring reported CMP at baseline (HUNT2), resulting in a study population of 4,742 offspring available for follow-up on risk of CMP. The complete inclusion and exclusion process is shown in Fig 1.

### Outcome measure

The participants were asked to complete a questionnaire that included items on musculoskeletal pain adopted from the Standardized Nordic Questionnaire [23]. These questions have acceptable reliability and validity for upper limb and neck pain [24], and are suggested to have a high utility in screening and surveillance [25]. In both HUNT2 and HUNT3 the participants were asked the following question about CMP:”During the last year, have you had pain and/or stiffness in your muscles and joints that lasted for at least three consecutive months?”. The response options were “yes” and “no”, and those who reported CMP were also asked to indicate if the pain had led to reduced leisure time activity (response options: “yes”, and “no”) or reduced their work ability (response options: “no”, “to some extent”, “considerably”, or “don’t know”). Offspring who answered “yes” to the question on reduced leisure time activity and/or reported work ability to be reduced “to some extent” or “considerably” were classified as having “activity-interfering CMP”.

### Exposure measures

Information on parental CMP was obtained from the same question as described above, and based on this we constructed a variable with four mutually exclusive categories reflecting parental reporting of CMP: “none”, “mother”, “father”, and “both parents”. As for offspring, we also constructed a variable indicating activity-interfering CMP in parents, with the same categories as for overall CMP: “none”, “mother”, “father”, and “both parents”.

Leisure time physical activity was assessed by the question “How much of your leisure time have you been physically active during the last year? (Think of a weekly average for the year. Your commute to work counts as leisure time)”. The participants reported number of hours of either light (no sweating or heavy breathing) or hard (sweating and heavy breathing) activity using the response options “none”, “less than 1 hour”, “1–2 hours”, and “3 or more hours” for each type of activity. Based on this information, we constructed a new variable with five categories combining information on light and hard activity: 1) “inactive” (no light or hard activity), 2) “low activity” (<3 hours light and no hard activity), 3) “moderate activity” (≥3 hours light and/or <1 hour hard activity), 4) “high activity” (any light and ≥1 hour hard activity), and 5) “unknown”. In the combined analyses of parental CMP and offspring physical activity the categories “inactive” and “low activity” were collapsed into one category labeled “low activity”.

Standardized measurements of body height (to the nearest centimeter) and body weight (to the nearest half kilogram) were obtained at the clinical examination. BMI was calculated as weight divided by the square of height (kg/m^2^), and the participants were then classified into four BMI groups according to the cut-off points suggested by the World Health Organization [26]: underweight (BMI <18.5 kg/m^2^), normal weight (BMI 18.5–24.9 kg/m^2^), overweight (BMI 25.0–29.9 kg/m^2^), and obese (BMI ≥30.0 kg/m^2^). Participants in HUNT3 were also asked about their height and weight at 18 years of age. Based on the above information we also classified offspring into normal weight (BMI <25 kg/m^2^) and overweight/obese (BMI ≥25 kg/m^2^) at both baseline (i.e., HUNT2) and at age 18 years.

### Statistical analysis

A Poisson regression model was used to estimate relative risk (RR) of CMP in offspring associated with parental CMP. We also assessed the combined effect of parental CMP and offspring leisure time physical activity, as well as of parental CMP and offspring BMI, on risk of CMP in offspring. Precision of RR was assessed by 95% confidence interval (CI). All standard errors were adjusted for within-family clustering (i.e., siblings) using the vce(cluster) option in Stata, treating observations between families as independent and within families as dependent, and thus avoiding inflated precision of the estimated associations [27].

The possible difference between maternal and paternal associations was evaluated using paternal CMP as the reference category in the regression model. Possible statistical interaction (i.e., departure from additivity) between mother-offspring and father-offspring associations was estimated as relative excess risk due to interaction (RERI). We calculated RERI estimates with 95% CIs from the following equation: RERI = RR_both parents_−RR_mother_−RR_father_ + 1 [28], i.e., RERI >0 indicate a synergistic effect beyond an additive effect. This approach was also used to assess possible interaction between parental pain and offspring physical activity, as well as between parental pain and offspring BMI. Additionally, statistical interaction was also assessed on a multiplicative scale by a likelihood ratio test of a product term in the model (without cluster-adjusted standard errors) as well as in analyses stratified by offspring physical activity and BMI categories (data not shown).

The main analyses were adjusted for possible confounding by offspring sex (male, female), age (continuous), BMI (“underweight”, “normal weight”, “overweight”, “obese”, or “unknown”), leisure time physical activity (“inactive”, “low activity”, moderate activity”, “high activity” or “unknown”), physical work demands (“mostly sedentary”, “much walking”, “much walking and lifting”, “heavy physical work”, or “unknown”), education (“<10 years”, “10–12 years”, “≥13 years”, or “unknown”), and psychological well-being (“satisfied”, “somewhat satisfied”, “dissatisfied”, or “unknown”). Analysis of the combined associations of parental pain and offspring physical activity or BMI did not include adjustment for the variable under study.

All statistical tests were two-sided, and all analyses were conducted using Stata for Windows, V.11.0 (StataCorp LP, Texas, USA).

## Results

In this prospective family-linkage study of 4,742 parent-offspring trios (2,592 daughters and 2,150 sons), a total of 1,700 offspring (35.8%) developed CMP during the follow-up period of approximately 11 years. The mean age at baseline was 33.3 (8.2) years among daughters and 35.1 (8.4) years among sons. Additional baseline characteristics of the study population are presented in Table 1.

**Table 1:** Baseline characteristics of the study population at HUNT2.

Variables		Daughters	Sons	Mothers	Fathers
Participants, no.		2,592	2,150	4,742	4,742
Age, mean (SD)		33.3 (8.2)	35.1 (8.4)	60.7 (9.9)	64.1 (10.2)
Categories of body mass index^a^					
	Normal weight, % (no.)	60.4 (1,545)	41.6 (887)	28.7 (1,354)	27.5 (1,301)
	Overweight, % (no.)	29.9 (765)	48.7 (1,039)	43.5 (2,052)	54.5 (2,579)
	Obese, % (no.)	8.9 (227)	9.8 (209)	27.1 (1,278)	17.1 (810)
	Unknown, % (no.)	0.8 (20)	0.3 (7)	0.7 (31)	0.8 (39)
Leisure time of physical activity^b^					
	Inactive, % (no.)	2.8 (73)	6.1 (130)	8.0 (381)	7.6 (363)
	Low activity, % (no.)	25.4 (659)	19.2 (413)	37.4 (1,771)	26.3 (1,246)
	Moderate activity, % (no.)	35.3 (915)	32.2 (692)	26.3 (1,247)	33.2 (1,574)
	High activity, % (no.)	35.3 (916)	41.5 (892)	9.7 (461)	19.9 (944)
	Unknown, % (no.)	1.1 (29)	1.1 (23)	18.6 (882)	13.0 (615)
Chronic musculoskeletal pain^c^					
Any pain, % (no.)		^e^	^e^	56.9 (2,699)	52.0 (2,464)
Activity interfering pain^d^, % (no.)		^e^	^e^	51.5 (2,171)	46.5 (1,981)

SD: standard deviation.

^a^Categories defined by WHO. The category “underweight” is not included.

^b^“Inactive” defined as no light or hard activity, “low” defined as <3 hours light and no hard activity, “moderate” defined as ≥3 hours light and/or <1 hour hard activity, and “high” defined as any light and ≥1 hour hard activity.

^c^Pain with duration ≥3 months during the last year at any location.

^d^Pain that interfere with work ability and/or leisure time activity.

^e^Offspring with pain at baseline (HUNT2) were excluded from the analyses.

Table 2 shows the RRs for CMP and activity-interfering CMP in offspring associated with parental CMP. Both maternal (RR: 1.26, 95% CI: 1.03, 1.55) and paternal (RR: 1.29, 95% CI: 1.06, 1.57) CMP was associated with increased risk of offspring CMP, with no difference in the strength of the association between parents (P_difference_ = 0.78). The risk of CMP was not stronger if both parents reported CMP (RR: 1.29, 95% CI: 1.06, 1.57), which was also reflected in the estimates of RERI (-0.28, 95% CI: -0.66, 0.09). Offspring risk of activity-interfering CMP was more strongly associated with maternal (RR: 1.3842, 95% CI: 1.13, 1.68) than paternal (RR: 1.08, 95% CI: 0.86, 1.35) CMP (P_difference_ = 0.02). These associations remained largely similar when the analysis was restricted to parental activity-interfering CMP, with RRs of 1.42 (95% CI: 1.16, 1.75) and 1.04 (95% CI: 0.82, 1.32), respectively (P_difference_ = 0.01). As indicated by the estimates of RERI there was no statistical evidence of a synergistic effect of CMP in both vs. only one parent (-0.02, 95% CI: -0.35, 0.38).

**Table 2:** Risk for offspring chronic musculoskeletal pain associated with chronic musculoskeletal pain in one or both parents.

		Any offspring CMP				Offspring activity-interfering CMP			
Variables		Case/Non-case	RR^a^	RR^b^ (95% CI)	RERI (95% CI)	Case/Non-case	RR^a^	RR^b^ (95% CI)	RERI (95% CI)
Any parental CMP									
	No CMP	320/735	1.00	1.00 (Ref.)		137/735	1.00	1.00 (Ref.)	
	Maternal	439/744	1.28	1.26 (1.03, 1.55)		209/744	1.38	1.38 (1.13, 1.68)	
	Paternal	337/613	1.31	1.30 (1.04, 1.62)		133/613	1.11	1.08 (0.86, 1.35)	
	Both parents	578/885	1.30	1.29 (1.06, 1.57)	-0.28 (-0.65, 0.09)	268/885	1.47	1.40 (1.15, 1.69)	-0.07 (-0.40, 0.26)
Parental activity-interfering CMP									
	No CMP	320/735	1.00	1.00 (Ref.)		137/735	1.00	1.00 (Ref.)	
	Maternal	353/595	1.27	1.25 (1.01, 1.56)		176/595	1.43	1.42 (1.16, 1.75)	
	Paternal	263/495	1.26	1.25 (0.99, 1.57)		103/495	1.07	1.04 (0.82, 1.32)	
	Both parents	393/569	1.36	1.35 (1.09, 1.67)	-0.15 (-0.54, 0.24)	188/569	1.56	1.48 (1.21, 1.81)	-0.02 (-0.35, 0.38)

CMP: chronic musculoskeletal pain, CI: confidence interval, RERI: relative excess risk due to interaction, RR: relative risk.

^a^Adjusted for offspring age (continuous) and sex in HUNT2.

^b^Adjusted for offspring factors in HUNT2; age (continuous), sex, body mass index (underweight, normal weight, overweight, obese, or unknown), leisure time physical activity (inactive, low, moderate, high, or unknown), physical work demands (mostly sedentary, much walking, much walking and lifting, heavy physical work, or unknown), psychological well-being (satisfied, somewhat satisfied, dissatisfied, or unknown), and education (<10 years, 10–12 years, ≥13 years, or unknown).

Table 3 shows the combined effect of parental CMP and offspring leisure time physical activity on offspring risk of activity-interfering CMP. Offspring with CMP present in both parents and who reported a low level of physical activity had a RR of 1.82 (95% CI: 1.32, 2.52) compared to offspring with high physical activity and no parental CMP. Offspring with CMP present in both parents, but who reported a high level of physical activity had a RR of 1.32 (95% CI: 0.95, 1.84). Although the offspring risk was higher among those with low physical activity, the estimate of RERI (0.24, 95% CI: -0.32, 0.79) indicated no synergistic effect between parental CMP and offspring physical activity. The analyses of parental-offspring CMP stratified by high, moderate, or low level of offspring physical activity showed that offspring where both parents reported CMP had RRs of 1.34 (95% CI:0.96, 1.86), 1.50 (95% CI:1.05, 2.13), and 1.43 (95% CI:1.05, 1.94), respectively (data not shown). We did not observe any statistical interaction (i.e., departure from a multiplicative effect) between parental pain and offspring physical activity (P = 0.15).

**Table 3:** Risk for offspring chronic musculoskeletal pain associated with the combined effect of parental chronic musculoskeletal pain and offspring leisure time physical activity.

		Offspring activity-interfering CMP		
Variables		High leisure time physical activity	Moderate leisure time physical activity	Low leisure time physical activity
No CMP				
	Cases/non-cases	49/301	38/239	48/188
	Age-adjusted RR	1.00	0.96	1.40
	RR^a^	1.00 (Ref.)	0.91 (0.62, 1.36)	1.29 (0.89, 1.87)
Any CMP, one parent				
	Case/non-case	215/894	241/780	202/659
	Age-adjusted RR	1.36	1.61	1.56
	RR^a^	1.30 (0.97, 1.75)	1.51 (1.13, 2.03)	1.38 (1.03, 1.87)
Any CMP, both parents				
	Case/non-case	86/352	82/307	96/217
	Age-adjusted RR	1.39	1.45	2.12
	RR^a^	1.32 (0.95, 1.84)	1.34 (0.96, 1.86)	1.82 (1.32, 2.50)

CMP: chronic musculoskeletal pain, CI: confidence interval, RR: relative risk.

^a^Adjusted for offspring factors in HUNT2; age (continuous), sex, body mass index (underweight, normal weight, overweight, obese, or unknown), physical work demands (mostly sedentary, much walking, much walking and lifting, heavy physical work, or unknown), psychological well-being (satisfied, somewhat satisfied, dissatisfied, or unknown), and education (<10 years, 10–12 years, ≥13 years, or unknown).

A similar pattern, but with somewhat stronger associations, where observed for the combined effect of parental CMP and offspring BMI (Table 4). Compared to normal weight offspring of pain-free parents, obese offspring of pain-afflicted parents had a RR of 2.33 (95% CI: 1.68, 3.24) while normal weight offspring of pain-afflicted parents had a RR of 1.32 (95% CI: 1.00, 1.74). An additional analysis adjusted for parental BMI did not alter the results (RRs of 2.29 [1.63, 3.20], and 1.32 [1.00, 1.75], respectively). There was weak evidence of a synergistic effect of parental CMP and offspring obesity on risk of offspring CMP with RERI of 0.88 (95% CI: 0.03, 1.73). The analyses of parental-offspring CMP stratified by offspring BMI showed that offspring where both parents reported CMP had RRs of 1.30 (95% CI: 1.03, 1.70) for normal weight offspring, 1.34 (95% CI: 1.01, 1.79) for overweight offspring, and 2.17 (95% CI: 1.22, 3.85) for obese offspring (data not shown). There was no statistically significant interaction on a multiplicative scale between parental pain and offspring physical activity (P = 0.51).

**Table 4:** Risk for offspring chronic musculoskeletal pain associated with the combined effect of parental chronic musculoskeletal pain and offspring body mass index.

		Offspring activity-interfering CMP		
Variables		Normal weight	Overweight	Obese
No CMP				
	Cases/non-cases	67/399	57/265	11/57
	Age-adjusted RR	1.00	1.30	1.15
	RR^a^	1.00 (Ref.)	1.24 (0.91, 1.70)	1.12 (0.63, 1.98)
Any CMP, one parent				
	Case/non-case	282/928	282/928	87/254
	Age-adjusted RR	1.36	1.66	1.75
	RR^a^	1.34 (1.04, 1.71)	1.57 (1.23, 2.02)	1.60 (1.19, 2.15)
Any CMP, both parents				
	Case/non-case	113/454	113/349	41/71
	Age-adjusted RR	1.37	1.78	2.57
	RR^a^	1.32 (1.00, 1.74)	1.67 (1.27, 2.20)	2.33 (1.68, 3.24)

CMP: chronic musculoskeletal pain, CI: confidence interval, RR: relative risk.

^a^Adjusted for offspring factors in HUNT2; age (continuous), sex, leisure time physical activity (inactive, low, moderate, high, or unknown), physical work demands (mostly sedentary, much walking, much walking and lifting, heavy physical work, or unknown), psychological well-being (satisfied, somewhat satisfied, dissatisfied, or unknown), and education (<10 years, 10–12 years, ≥13 years, or unknown).

In supplementary analyses we further explored the combined effect of BMI and parental CMP using information on offspring BMI at age 18 years (data not shown). Offspring were both parents reported CMP had a RR of 2.01 (95% CI: 1.17, 3.48) if they had a BMI ≥25 kg/m^2^ and a RR of 1.31 (95% CI: 0.89, 1.93) if the BMI was <25 kg/m^2^ compared to normal weight offspring of pain-free parents.

## Discussion

In this prospective family-linkage study we found that parental CMP was positively associated with risk of CMP in the adult offspring. There were no clear differences between father-offspring and mother-offspring associations for any CMP. However, when the analyses were restricted to offspring risk of activity-interfering CMP, the association was stronger for maternal than paternal CMP. The results of the combined analyses showed that offspring risk of activity-interfering CMP associated with parental CMP was somewhat higher among offspring who reported a low level of leisure time physical activity compared to a high physical activity level. However, the results did not suggest association above additivity. The adverse association of parental CMP with offspring activity-interfering CMP was somewhat stronger among overweight and obese offspring compared to normal weight offspring, suggesting that offspring BMI may modify parent-offspring association of CMP.

Parental pain has been associated with increased occurrence of offspring pain in childhood and adolescence in some studies [1, 29, 30], but not in others [31, 32]. Inconsistent results could be explained by the different definitions of (chronic) pain, the type and pain sites under study, and the different methods for pain reporting (i.e., own reporting vs. from other family members) [31, 33]. Only a few studies [3, 31, 32] have used independent information on pain from both parents and offspring; however, it has been argued that this is a crucial advantage in studies of intergenerational associations of pain to avoid bias and misclassification in pain reporting [34]. Based on independent data on pain from parents and offspring, we have in a recent cross-sectional study showed that presence of parental CMP was associated with increased occurrence of CMP in the adult offspring [35]. The current study extends on the abovementioned findings, showing that presence of parental CMP is prospectively associated with risk of CMP in the adult offspring.

The inter-generational transfer of CMP is likely related to both genetic factors and environmental influences. A few studies have pointed at specific genetic markers that are involved in the etiology of chronic pain [5], disabling low back pain [36] and fibromyalgia [33, 37]. However, leisure time physical activity [38], obesity [38], socioeconomic status [39], and psychological wellbeing [40] are factors that track across generations, and these factors have also been associated with development of CMP [9–14, 41, 42]. Thus, shared lifestyle and societal factors could contribute to the association of CMP between parents and their adult offspring; however, adult offspring are less likely to share environmental factors with their parents than are children and adolescents. Importantly, the current study indicates that the lifestyle of the adult offspring modify the parent-to-offspring transfer of CMP.

Both physical inactivity and obesity have been related to increased risk of CMP [9–14], but whether lifestyle factors such as obesity and inactivity could modify the association between parental and offspring CMP has not previously been examined. Although the association between parental CMP and offspring risk of activity-interfering CMP was higher in offspring who reported low level of leisure time physical activity compared to moderate or high levels, there was no evidence of synergistic effects above what could be expected from the additive effect of each risk factor. However, inconsistent associations between physical activity and CMP has been reported [43], suggesting that both low and high levels of physical activity can increase the risk of CMP. If such differential associations with physical activity exist this could mask a possible modifying effect of physical activity in our analyses. There was evidence that offspring BMI could be an effect modifier on the parent-offspring association of CMP, with somewhat stronger associations among offspring who were classified as overweight or obese than those who were normal weight. It has been suggested that inter-individual differences in pain sensitivity and endogenous pain-inhibitory capacity could reflect variations in the inherent susceptibility for chronic pain [44, 45], but that a triggering insult or exposure is required for the development of chronic pain [37, 46]. This could imply that a possible genetic predisposition for CMP [47, 48] has a higher penetrance among offspring with a physically inactive lifestyle and/or who are overweight or obese. Moreover, it has been shown that factors early in life is associated with risk of pain [49], and in the present study, a BMI ≥25 kg/m^2^ at the age of 18 years was associated with a particularly detrimental effect of parental pain compared to those who reported a BMI <25 kg/m^2^ at 18 years. Although this could imply that adiposity in adolescence and early adulthood could modify the risk of CMP among persons with a heritable component of chronic pain, it cannot rule out the possibility that such factors are a common cause of both parental and offspring pain.

We observed that mother-offspring and father-offspring associations were equally related to offspring risk of any CMP, whereas the risk of more severe and activity interfering CMP was more strongly related to maternal than paternal CMP. Information bias arising from non-paternity [50], where the biological father is not the same as the reported father could result in a weaker association through the paternal line compared with the maternal line. A recent study from the same population estimated the influence of various non-paternity rates on parent-offspring associations in continuously measured cardiovascular disease risk factors [35]. A stronger maternal-offspring than paternal-offspring association for some of the risk factors were cancelled out when assuming non-paternity rates of 3–5%. The possible influence of non-paternity was not assessed in the current study due to the nature of the data (i.e. binary exposures and outcomes). However, the somewhat differential associations with mother’s and father’s CMP could reflect non-paternity, but could also imply that the maternal influence is higher than paternal influences, particularly through environmental and behavioral factors [50]. Studies have shown that children of parents who display pain behavior learn to display similar pain behavior and are also more likely to report pain [6, 8, 51].

Important strengths of the current study include the prospective design, excluding offspring with CMP at baseline, the registry based information on family relations, the information on CMP obtained from parents and offspring independently and at different time points, and the inclusion of adult offspring who predominantly will not share household with their parents. Moreover, we were able to adjust for several offspring characteristics that could confound the parent-offspring associations of CMP, such as age [11], BMI [14], leisure time physical activity [12], physical work demands [52], psychological well-being [53, 54], and education [5, 55]. Nevertheless, possible residual confounding due to unknown or unmeasured factors cannot be ruled out.

There are some limitations that should be considered when interpreting the results of this study. Information on CMP was only reported at baseline and at follow-up 11 years later, with no information on possible changes in CMP during the follow-up period. Thus, some offspring could have experienced intermittent CMP that was not captured upon participation in the health survey. However, it is not likely that this was differential between offspring with parents who reported CMP and those who did not. Similarly, information on leisure time physical activity and BMI was only assessed at baseline, with no information on possible changes throughout the follow-up period. The questions about leisure time physical activity used in this study has been validated against more objective measures of fitness and activity, such as VO_2_max and ActiReg in a subsample of young men [56]. Although the questionnaire has been reported to have good repeatability and provide useful measures of leisure time physical exercise, subjective interpretations of the activity questions could have influenced the results. Moreover, a premise for inclusion into this study was that the mother, father and offspring all had to participate in the health survey. This may have resulted in a somewhat selected and more health conscious sample than the general population. However, it is disputable whether representativeness is a prerequisite for making valid risk assessments in epidemiological studies [57].

In conclusion, this prospective study shows that parental CMP is positively associated with risk of CMP in the adult offspring. There was no clear evidence of effect modification by offspring leisure time physical activity, but maintenance of normal body weight may reduce the risk of CMP in offspring of pain-afflicted parents. Community-based measures aimed at reducing the incidence of CMP should therefore emphasize the importance of a healthy lifestyle, especially in families with a history of CMP.
